# Hematological disruptions in HIV-positive individuals with cardiac arrhythmias: a focus on coagulation and inflammation – a narrative review

**DOI:** 10.1097/MS9.0000000000003714

**Published:** 2025-08-08

**Authors:** Emmanuel Ifeanyi Obeagu, Olga G. Goryacheva, Mekhman Mamedov

**Affiliations:** a Department of Biomedical and Laboratory Science, Africa University, Mutare, Zimbabwe; b Department of Polyclinic Therapy, Perm State Medical University named after Academician E.A. Wagner, Ministry of Health of the Russian Federation, Perm, Russia; c Head Department for Secondary Prevention of the Chronic Non-Infectious Diseases, National Research Centre for Therapy and Preventive Medicine, Moscow, Russia

**Keywords:** cardiac arrhythmias, coagulation, hematological disruptions, HIV, inflammation

## Abstract

HIV infection is linked to hematological disruptions, notably coagulation abnormalities and chronic inflammation, which contribute to the rising prevalence of cardiac arrhythmias in this population. Elevated fibrinogen, factor VIII, and von Willebrand factor levels promote a hypercoagulable state, while cytokines like IL-6 and TNF-α drive persistent inflammation. These factors interact to induce cardiac electrical remodeling, increasing susceptibility to arrhythmias such as atrial fibrillation and ventricular tachycardia. Additionally, HIV-related inflammation accelerates atherosclerosis, further elevating cardiovascular risk. Some antiretroviral therapies exacerbate prothrombotic tendencies, underscoring the importance of close hematological monitoring. Recognizing and addressing these disruptions is essential for preventing arrhythmias and improving outcomes in HIV-positive individuals.

## Introduction

Human immunodeficiency virus (HIV) is now recognized as a chronic, multisystem condition, with significant extrapulmonary, cardiovascular, renal, and neurological manifestations beyond its classical immunodeficiency profile. This expanded understanding is critical when addressing comorbidities such as cardiac arrhythmias in people living with HIV (PLWH)[1]. HIV continues to be a global public health challenge, with significant advances in antiretroviral therapy (ART) leading to improved life expectancy and quality of life for many patients[2]. However, HIV-infected individuals face a higher risk of developing a range of comorbidities, including cardiovascular diseases. Among the cardiovascular complications, cardiac arrhythmias have emerged as a prominent concern, often exacerbated by HIV-related hematological disruptions. These arrhythmias can significantly impact the health and prognosis of HIV-positive individuals, contributing to increased morbidity and mortality^[3,4]^. Hematological alterations in HIV-positive individuals are multifactorial and can result from both direct viral effects and the consequences of prolonged immune activation. One of the key features of HIV infection is the persistent inflammatory state, which can lead to endothelial dysfunction, alterations in platelet function, and a dysregulated coagulation cascade. These changes increase the likelihood of thromboembolic events, contributing to the development of cardiac arrhythmias. Additionally, the use of antiretroviral drugs, especially those that alter lipid metabolism and endothelial function, can exacerbate these hematological abnormalities, further increasing the risk of arrhythmias^[5–8]^. Coagulation disorders in HIV-infected individuals are primarily driven by chronic immune activation and the release of pro-inflammatory cytokines. HIV infection leads to elevated levels of fibrinogen, factor VIII, and von Willebrand factor (vWF), all of which promote a prothrombotic environment. This hypercoagulable state increases the risk of venous thromboembolism, stroke, and myocardial infarction. Furthermore, HIV-associated inflammation also affects the myocardial tissue, contributing to structural and electrical remodeling of the heart, which predisposes to arrhythmias. These arrhythmias can manifest as atrial fibrillation, ventricular tachycardia, and other rhythm disturbances, which are associated with an increased risk of sudden cardiac death in HIV-positive patients^[9–11]^.

**HIGHLIGHTS**

- HIV-positive individuals with cardiac arrhythmias often exhibit hypercoagulability, increasing thrombotic risk.
- Chronic inflammation exacerbates endothelial dysfunction and promotes arrhythmogenic conditions.
- Coagulation abnormalities, including elevated D-dimer levels, are frequent.
- Inflammatory cytokines (e.g., IL-6, TNF-α) intensify cardiac and hematologic disturbances.
- Antiretroviral therapy may influence both coagulation cascades and inflammatory profiles.

In addition to coagulation abnormalities, chronic inflammation plays a significant role in the pathogenesis of arrhythmias in HIV patients. Elevated levels of inflammatory markers, such as C-reactive protein (CRP), interleukin-6 (IL-6), and tumor necrosis factor-alpha (TNF-α), have been implicated in the electrical remodeling of the heart. These cytokines contribute to myocardial fibrosis, endothelial dysfunction, and changes in the ionic currents of cardiac myocytes, leading to arrhythmogenic foci. The inflammatory response also accelerates atherosclerosis, increasing the risk of ischemic heart disease, which further complicates the management of cardiac arrhythmias in this population^[12,13]^.

Studies have shown that the prevalence of cardiac arrhythmias in HIV-positive individuals is higher than in the general population, particularly among those with more advanced disease. This increased prevalence can be attributed to a combination of factors, including the direct effects of the virus on the heart, the toxic effects of antiretroviral medications, and the hematological disruptions mentioned earlier. HIV-associated arrhythmias often occur in individuals with additional risk factors, such as hypertension, diabetes, and dyslipidemia, further complicating the clinical picture. Given the growing recognition of the prevalence of arrhythmias in this patient population, it is crucial to identify early biomarkers for predicting arrhythmia risk and to develop appropriate management strategies^[14–16]^. The diagnosis and management of cardiac arrhythmias in HIV-positive individuals are challenging due to the complex interplay between viral infection, antiretroviral therapy, and hematological abnormalities. Clinicians must be aware of the increased risk of arrhythmias in this population and carefully monitor patients for signs and symptoms. Regular screening for coagulation imbalances, inflammatory markers, and cardiac function is essential to detect early signs of arrhythmias and implement timely interventions. Furthermore, individualized treatment approaches that consider the unique aspects of HIV infection, ART, and comorbidities are needed to optimize outcomes for HIV-positive individuals with cardiac arrhythmias^[16,17]^. While the role of coagulation and inflammation in the development of cardiac arrhythmias in HIV patients is becoming better understood, there is still a need for more comprehensive research to elucidate the exact mechanisms involved. Investigating the relationship between specific hematological markers, such as D-dimer, fibrinogen, and inflammatory cytokines, and the development of arrhythmias in HIV-infected individuals could lead to more effective diagnostic and therapeutic strategies. Moreover, there is a need for studies exploring how different classes of ART influence the risk of arrhythmias and how best to manage coagulation and inflammation in this context^[18,19]^.

## Aim

The aim of this review is to critically examine the hematological disruptions observed in HIV-positive individuals with cardiac arrhythmias, with a specific focus on coagulation abnormalities and inflammatory processes.

## Review methods

**Figure 1.**
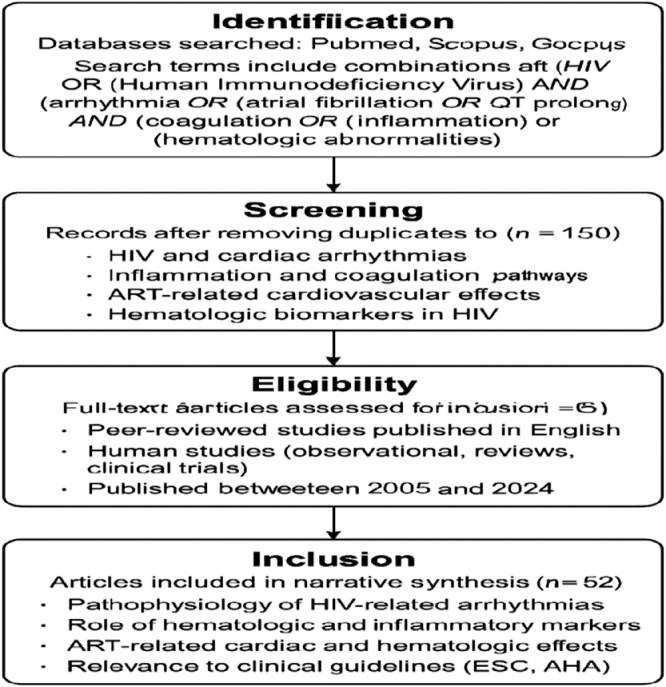
A simplified flow diagram of the literature search and selection process for this narrative review. The search spanned three major databases (PubMed, Scopus, and Google Scholar) and included studies focused on the intersection of HIV, cardiac arrhythmias, inflammation, coagulation, and hematologic markers published between 2005 and 2024.

This review adopted a narrative approach to synthesize current literature on hematological disruptions in HIV-positive individuals with cardiac arrhythmias, particularly emphasizing coagulation abnormalities and inflammatory pathways. The methodology involved a structured search of peer-reviewed articles, clinical reports, and review papers published between 2000 and 2024. Databases including PubMed, Google Scholar, Scopus, and Web of Science were extensively searched using relevant keywords such as *HIV, hematological profile, cardiac arrhythmias, coagulation disorders*, and *inflammation*. Boolean operators and Medical Subject Headings (MeSH) terms were employed to refine search results and enhance relevance. The inclusion criteria were studies that provided clinical, mechanistic, or epidemiological insights into hematological changes in HIV-infected individuals, with a focus on their relationship to cardiovascular health and arrhythmogenic risk. Articles written in English and involving human subjects were prioritized. Excluded materials included non-peer-reviewed articles, case reports lacking broader clinical applicability, and studies that focused exclusively on pediatric populations or individuals without cardiac manifestations. Preference was given to high-quality studies, systematic reviews, and research with large sample sizes or significant clinical impact. Data extracted from selected studies were thematically analyzed and categorized into major focus areas, including coagulation abnormalities, inflammation-mediated cardiac effects, and their implications for clinical management. This method enabled the integration of diverse perspectives from immunology, hematology, and cardiology. By consolidating findings across disciplines, the review provides a comprehensive overview of the complex interplay between HIV-related hematological disorders and cardiac arrhythmias, supporting informed decision-making in clinical practice and identifying areas for future research (Fig. 1).

### Limitations of the review

While this review provides a comprehensive synthesis of the current knowledge on hematological disruptions and arrhythmia risk in HIV-positive individuals, several limitations must be acknowledged. First, the included studies exhibit significant heterogeneity in terms of study design, patient populations, outcome measures, and biomarker assessment methods, which may affect the generalizability of the findings. Second, much of the available evidence is derived from observational studies, which are inherently subject to confounding variables and cannot establish causality. The lack of randomized controlled trials (RCTs) specifically investigating the interplay between coagulation, inflammation, and arrhythmias in the context of HIV limits the strength of the conclusions that can be drawn. Finally, publication bias and the underrepresentation of low-resource settings may further constrain the applicability of the findings across diverse HIV populations.

### Hematological disruptions in HIV and their impact on cardiac arrhythmias

HIV infection, a global public health concern, has multifaceted effects on the cardiovascular system. While advancements in ART have significantly improved the life expectancy of individuals living with HIV, these individuals continue to face an elevated risk of developing cardiovascular complications, particularly cardiac arrhythmias. One of the key contributors to the increased risk of arrhythmias is the presence of hematological disruptions, which include altered coagulation profiles and chronic inflammation. These disruptions not only contribute to the pathophysiology of arrhythmias but also increase the morbidity and mortality associated with cardiovascular events in HIV-positive individuals.

### Coagulation abnormalities in HIV

Coagulation disruptions are a hallmark of HIV infection and are influenced by both the viral infection itself and the antiretroviral therapies that patients often rely on for treatment. HIV-induced chronic inflammation triggers the release of pro-inflammatory cytokines such as TNF-α and IL-6, which in turn increase the levels of clotting factors like fibrinogen, factor VIII, and vWF. This hypercoagulable state predisposes individuals to thrombotic events such as deep vein thrombosis, pulmonary embolism, and ischemic stroke, which, in turn, elevate the risk of arrhythmias. The blood clotting cascade also affects the heart’s electrical conductivity, contributing to arrhythmic changes by facilitating myocardial injury and fibrosis. One particular consequence of coagulation abnormalities in HIV-infected individuals is the development of atrial fibrillation (AF), a common arrhythmia that can lead to stroke and other complications. AF is thought to occur due to the combination of increased blood viscosity, endothelial dysfunction, and structural remodeling of the atria, driven by the inflammatory milieu associated with chronic HIV infection. The risk of thromboembolic events such as stroke in individuals with AF is exacerbated in HIV-infected patients, particularly in those with low CD4 counts and high viral loads^[20–22]^.

### Inflammatory mechanisms and cardiac arrhythmias

Elevated levels of IL-6, TNF-α and CRP contribute to myocardial fibrosis, which alters the conduction system of the heart and increases the susceptibility to arrhythmias. Additionally, this inflammatory state promotes endothelial dysfunction, which impairs the vascular response to stress and increases the risk of ischemic heart disease – a key contributor to arrhythmic conditions. Moreover, inflammation in HIV-positive individuals accelerates atherosclerosis, a condition characterized by the buildup of plaque in the arterial walls, which can reduce blood flow to the heart and provoke arrhythmic disturbances. The process of atherosclerosis involves chronic inflammation and endothelial damage, which leads to the formation of unstable plaques and increases the risk of sudden cardiac events. The interaction between these inflammatory and coagulation pathways ultimately creates a favorable environment for the development of arrhythmias, particularly in individuals with additional risk factors such as hypertension, diabetes, and dyslipidemia (Table 1)^[15,16,23,24]^.

**Table 1:** Clinical biomarkers associated with arrhythmia risk in HIV-positive individuals

Biomarker	Category	Clinical Role in arrhythmia	Association with HIV
D-dimer	Coagulation marker	Indicates fibrinolysis and thrombotic activity; elevated in atrial fibrillation (AF)	Frequently elevated in HIV; marker of systemic hypercoagulability
Fibrinogen	Coagulation marker	Promotes clot formation; elevated levels linked with AF and stroke	HIV infection induces chronic fibrinogen elevation
von Willebrand Factor (vWF)	Endothelial dysfunction	Marker of endothelial injury; associated with increased arrhythmic burden	Elevated in chronic HIV inflammation and endothelial activation
Interleukin-6 (IL-6)	Pro-inflammatory cytokine	Promotes electrical and structural remodeling in atrial myocardium	IL-6 levels are persistently elevated in ART-treated and untreated HIV patients
Tumor Necrosis Factor-α (TNF-α)	Pro-inflammatory cytokine	Induces ion channel dysfunction and myocardial fibrosis	Upregulated in HIV; correlates with immune activation and vascular inflammation
C-reactive Protein (CRP)	Acute phase reactant	Associated with increased risk of AF and sudden cardiac death	Elevated in HIV; reflects chronic systemic inflammation
IL-1β	Pro-inflammatory cytokine	Contributes to myocardial fibrosis and electrical conduction disturbances	Upregulated in chronic HIV infection and co-infections
NT-proBNP	Cardiac stress marker	Elevated in heart strain and predictive of arrhythmia-related events	Increased in HIV-related myocardial dysfunction and inflammation
CD4 Count	Immunological marker	Low counts correlate with higher inflammatory burden and cardiovascular risk	Key marker of disease progression and immune suppression
HIV Viral Load	Virological marker	High viral loads associated with increased inflammatory and coagulative activity	Predicts systemic immune activation and cardiovascular complications

### Antiretroviral therapy and its impact on hematological parameters

Antiretroviral therapy, the cornerstone of HIV treatment, has made significant strides in prolonging life expectancy among HIV-infected individuals. However, ART also introduces its own set of risks, particularly regarding hematological disruptions. Certain classes of ART, such as protease inhibitors and non-nucleoside reverse transcriptase inhibitors, have been associated with increased lipid levels, insulin resistance, and endothelial dysfunction, all of which contribute to cardiovascular risk. Additionally, these therapies can exacerbate the coagulation abnormalities seen in HIV-infected individuals, further elevating the risk of arrhythmias. The effects of ART on coagulation and inflammation can vary depending on the regimen used. For instance, protease inhibitors, while effective in controlling viral replication, are known to have prothrombotic effects and can worsen the hypercoagulable state in HIV patients. On the other hand, integrase inhibitors, which may less likely to influence coagulation, are not without their own set of side effects, such as increased cardiovascular risk. This complex interplay between ART, coagulation, and inflammation requires careful management to reduce the risk of arrhythmias and other cardiovascular complications^[25–28]^.

### The pathophysiology of arrhythmias in HIV-infected individuals

The pathophysiology of arrhythmias in HIV-infected individuals is multifactorial, with hematological disruptions playing a critical role. Coagulation abnormalities and persistent inflammation both contribute to myocardial remodeling, which is the structural basis for arrhythmogenesis. In the presence of a prothrombotic state, clot formation can lead to ischemia or infarction, both of which can disrupt the normal electrical activity of the heart and result in arrhythmias such as ventricular tachycardia or atrial fibrillation. Furthermore, the immune response triggered by HIV can cause myocardial inflammation and fibrosis, altering the electrical properties of the heart and making it more prone to arrhythmias. The pro-inflammatory cytokines and coagulation factors increase myocardial excitability, reduce the effectiveness of the cardiac conduction system, and lead to the development of arrhythmic foci that can trigger sustained arrhythmias. These processes underscore the need for early detection of hematological disruptions and timely management to prevent the development of life-threatening arrhythmias (Table 2)^[29–31]^.

**Table 2:** Pathophysiological Links of Arrhythmia Risk in HIV-Positive Individuals

Pathophysiological Mechanism	Description	Contribution to Arrhythmia	Relevance in HIV
Chronic Inflammation	Persistent elevation of cytokines (e.g., IL-6, TNF-α, IL-1β)	Promotes atrial and ventricular remodeling, fibrosis, and autonomic dysfunction	Common in untreated and ART-treated HIV; linked to systemic immune activation
Coagulation Abnormalities	Increased D-dimer, fibrinogen, vWF levels; endothelial dysfunction	Hypercoagulability promotes microvascular thrombosis and ischemia-triggered arrhythmia	HIV induces a prothrombotic state through direct viral and immune-mediated mechanisms
Direct Viral Effects on Myocardium	HIV infects cardiac myocytes and induces mitochondrial dysfunction	Contributes to cardiomyopathy and electrical conduction abnormalities	HIV RNA and proteins have been detected in cardiac tissue
Autonomic Nervous System Dysfunction	Altered sympathetic/parasympathetic tone due to inflammation and neural injury	Leads to heart rate variability, QT prolongation, and arrhythmic susceptibility	Autonomic imbalance is common in chronic HIV infection
Myocardial Fibrosis and Remodeling	Fibrosis induced by inflammatory mediators and ischemia	Structural substrate for atrial fibrillation and ventricular tachycardia	MRI and autopsy studies reveal myocardial fibrosis in HIV patients
Atherosclerosis and Ischemia	Accelerated plaque formation due to inflammation and dyslipidemia	Ischemic injury predisposes to arrhythmogenic foci	Early-onset atherosclerosis is well documented in HIV
Adverse Effects of Antiretroviral Therapy (ART)	Some ARTs (e.g., protease inhibitors) contribute to metabolic and cardiac dysfunction	May prolong QT interval and alter conduction pathways	ART-related cardiotoxicity adds to arrhythmic risk
Electrolyte Imbalances	HIV and ART-associated renal dysfunction can lead to hypo/hyperkalemia, hypomagnesemia	Electrolyte shifts contribute to arrhythmic events, especially ventricular arrhythmias	Frequently seen in advanced disease or with opportunistic infections
Immunosuppression (Low CD4 Counts)	Weakened immune surveillance and high viral burden		

### Clinical implications and management strategies

The presence of hematological disruptions in HIV-positive individuals necessitates a comprehensive approach to managing cardiac arrhythmias. Early recognition of coagulation abnormalities and inflammation through regular screening for biomarkers such as fibrinogen, D-dimer, IL-6, and CRP is essential in identifying individuals at risk of arrhythmias. Moreover, individualized management strategies should be developed to optimize ART regimens, considering the potential effects on coagulation and inflammation, while also addressing other cardiovascular risk factors like hypertension, dyslipidemia, and smoking. In cases where arrhythmias are detected, management may include anticoagulation therapy to reduce the risk of thromboembolic events, as well as anti-inflammatory drugs to mitigate the inflammatory state. The use of rate or rhythm control medications, such as beta-blockers or antiarrhythmic drugs, may also be required to manage the arrhythmic events themselves. Close monitoring of hematological and cardiovascular parameters is essential to prevent complications and improve the long-term outcomes for HIV-positive individuals with arrhythmias^[31–33]^.

### Coagulation abnormalities and their role in cardiac arrhythmias

Coagulation abnormalities are a prominent hematological manifestation in individuals living with HIV, largely driven by chronic immune activation, systemic inflammation, and direct endothelial injury. These abnormalities contribute significantly to cardiovascular complications, including cardiac arrhythmias. In HIV-positive individuals, the balance between pro-coagulant and anticoagulant mechanisms is disrupted, favoring a hypercoagulable state. Elevated levels of pro-coagulant factors such as fibrinogen, D-dimer, tissue factor, and von Willebrand factor, alongside diminished levels of natural anticoagulants like protein C, protein S, and antithrombin III, create an environment conducive to thrombosis and subsequent cardiac disturbances. This prothrombotic milieu increases the risk of both microvascular and macrovascular thrombotic events, which can impair myocardial perfusion and promote myocardial ischemia. Ischemic conditions are well-known triggers of arrhythmogenic mechanisms, such as ectopic automaticity and reentry circuits, leading to arrhythmias like atrial fibrillation and ventricular tachyarrhythmias. Additionally, thrombotic occlusion in coronary or pulmonary vessels may induce acute strain on the myocardium, altering its electrophysiological properties and predisposing the heart to rhythm instability. Inflammation-induced platelet activation and endothelial dysfunction further exacerbate these risks by promoting vascular injury and fibrotic remodeling of cardiac tissue^[23,34]^. Moreover, the advent of highly active antiretroviral therapy (HAART) has paradoxically contributed to coagulation imbalance in some patients. Certain ART regimens, particularly protease inhibitors, have been linked to metabolic syndrome, dyslipidemia, and increased thrombogenic potential. These factors cumulatively raise the likelihood of thromboembolic events, which are frequently associated with the onset or worsening of arrhythmias. For instance, atrial fibrillation in HIV-positive individuals has been strongly associated with elevated D-dimer levels and inflammatory markers – suggesting a link between hypercoagulability and electrical instability of the heart. Effective management of coagulation abnormalities in HIV-infected patients, therefore, plays a crucial role in reducing the burden of cardiac arrhythmias and enhancing long-term cardiovascular outcomes[34].

### Inflammation as a contributor to cardiac arrhythmias in HIV

Chronic inflammation is a hallmark of HIV infection, persisting even in patients receiving effective ART. This persistent immune activation stems from ongoing viral replication, microbial translocation, co-infections, and immune dysregulation. In the cardiovascular context, inflammation plays a pivotal role in the development of structural and electrical abnormalities that predispose individuals to cardiac arrhythmias[35]. Inflammation contributes to arrhythmias by promoting myocardial fibrosis, altering ion channel expression, and impairing autonomic regulation of the heart. Fibrotic remodeling of the myocardium can disrupt normal conduction pathways and foster re-entrant circuits, creating a substrate for both atrial and ventricular arrhythmias. Furthermore, inflammatory cytokines can directly modulate the function of cardiac ion channels, particularly those governing sodium and potassium currents, leading to abnormal depolarization and repolarization. These electrophysiological disturbances heighten the risk of arrhythmogenic events such as premature beats, prolonged QT intervals, and sudden cardiac death[36]. The link between systemic inflammation and arrhythmias in HIV is further supported by epidemiological data showing higher rates of atrial fibrillation and ventricular arrhythmias in patients with elevated inflammatory markers. Additionally, the presence of co-infections such as cytomegalovirus (CMV) or hepatitis C virus (HCV), which are common in HIV populations, can amplify the inflammatory response, and exacerbate cardiac risk. Inflammatory pathways also overlap with coagulation cascades, creating a synergistic effect that further increases susceptibility to arrhythmias^[37,38]^.

### Integration with clinical guidelines: ESC and AHA/HRS perspectives on arrhythmia risk in HIV-positive patients

Current evidence underscores the necessity of aligning HIV care with evolving cardiovascular guidelines, particularly those concerning arrhythmia risk assessment and management. The European Society of Cardiology (ESC) 2020 guidelines on atrial fibrillation (AF) and the American Heart Association/American College of Cardiology/Heart Rhythm Society (AHA/ACC/HRS) 2019 guidelines on arrhythmias both provide robust frameworks for stratifying and managing arrhythmogenic risk. However, their application in HIV-positive populations remains underrepresented in clinical practice and research[39]. The ESC 2020 guidelines emphasize the importance of systemic inflammation, atrial remodeling, and prothrombotic states in the pathogenesis of arrhythmias – factors that are frequently present in people living with HIV (PLWH). These include elevated levels of D-dimer, interleukin-6 (IL-6), and C-reactive protein (CRP) – well-documented markers of immune activation in chronic HIV infection. Furthermore, the ESC recommends individualized thromboembolic risk assessment using the CHA₂DS₂-VASc score, which could be adapted in PLWH by incorporating HIV-related inflammatory markers to improve risk stratification[40].

Similarly, the AHA/ACC/HRS 2019 arrhythmia guidelines acknowledge the need to consider comorbid inflammatory and metabolic conditions in arrhythmia management. HIV infection, particularly in the presence of antiretroviral therapy (ART)-induced dyslipidemia, QT prolongation, or autonomic dysfunction, represents a clinically relevant context for arrhythmia surveillance. These guidelines support the use of continuous ECG monitoring and echocardiographic assessment in high-risk individuals – tools that could be deployed proactively in HIV cohorts exhibiting hematologic and inflammatory abnormalities^[39,40]^.

### Clinical implications and management strategies

The intersection of hematological disruptions, inflammation, and coagulation abnormalities in HIV-positive individuals with cardiac arrhythmias presents significant clinical challenges and necessitates a multidimensional management approach. These patients are often at increased risk of thromboembolic events, sudden cardiac death, and complications arising from both the infection and its treatment. Clinicians must therefore adopt a holistic strategy that includes vigilant cardiovascular monitoring, comprehensive hematological assessments, and early identification of arrhythmic symptoms. Routine screening for coagulation markers (e.g., D-dimer, fibrinogen) and inflammatory mediators (e.g., CRP, IL-6) is crucial to identifying patients at high risk for cardiovascular complications[41]. ART selection also plays a central role in the management strategy. Some ART regimens, particularly protease inhibitors, have been associated with increased cardiovascular risk due to their impact on lipid metabolism and endothelial function. Clinicians should consider cardiovascular profiles when initiating or adjusting ART regimens and aim to use agents with a more favorable cardiometabolic impact. Additionally, integrating cardioprotective therapies – such as beta-blockers, anticoagulants, and antiarrhythmic drugs – tailored to the individual’s risk profile can help manage both the electrical and structural abnormalities underlying arrhythmias^[42,43]^. Importantly, a multidisciplinary approach involving infectious disease specialists, cardiologists, and hematologists can enhance patient outcomes through coordinated care. Lifestyle interventions including smoking cessation, dietary improvements, regular physical activity, and adherence to medication regimens are also critical components of comprehensive care. With the increasing life expectancy of HIV-infected individuals, proactive management of cardiovascular and hematological health is essential to improving quality of life and reducing long-term morbidity and mortality associated with cardiac arrhythmias in this population. Continued research and the development of guidelines specific to this unique cohort are vital to optimizing care and outcomes^[44,45]^.

### Future directions and knowledge gaps

Despite growing recognition of the intersection between hematological disturbances, inflammation, and cardiac arrhythmias in HIV-positive individuals, several critical knowledge gaps remain. Addressing these limitations through focused research will be essential for improving cardiovascular outcomes in this population.

1. Need for prospective and longitudinal studies: Most current evidence is derived from cross-sectional or retrospective analyses. There is a pressing need for large-scale prospective cohort studies to clarify temporal relationships between inflammatory/coagulative biomarkers and arrhythmia onset in HIV-positive populations. These studies should ideally incorporate serial biomarker assessments and electrocardiographic monitoring[46].
2. Integration of biomarkers into clinical algorithms: Although markers such as D-dimer, IL-6, CRP, and NLR are elevated in HIV and associated with cardiovascular risk, they have not yet been fully incorporated into clinical risk stratification tools like the CHA₂DS₂-VASc score. Future research should focus on developing or refining risk prediction models that integrate both traditional cardiovascular risk factors and HIV-specific inflammatory or coagulative biomarkers[47].
3. ART-specific cardiovascular risk profiling: While antiretroviral therapy (ART) has dramatically improved survival, certain regimens (e.g., protease inhibitors, integrase strand transfer inhibitors) have been linked to adverse metabolic profiles and QT prolongation. Future studies must delineate the arrhythmogenic potential of specific ART agents and explore how cumulative ART exposure or regimen switching influences electrophysiological and hematologic parameters[46].
4. Underrepresentation in clinical guidelines: Current arrhythmia management guidelines (e.g., ESC 2020, AHA/ACC/HRS 2019) do not specifically address HIV as a modifier of arrhythmia risk. Advocacy for inclusion of HIV-specific recommendations in future iterations of these guidelines is warranted, especially in light of rising cardiovascular morbidity in aging HIV populations[47].
5. Personalized therapeutic approaches: With advancements in biomarker discovery and digital health monitoring, the potential exists for individualized cardiovascular surveillance and anticoagulation strategies tailored to the inflammatory and coagulation profile of HIV-positive patients. Further research into personalized medicine frameworks in this setting is highly encouraged[48].

## Conclusion

Hematological disruptions – particularly involving inflammation and coagulation – play a pivotal role in the pathophysiology of cardiac arrhythmias among people living with HIV. Chronic immune activation, endothelial dysfunction, and antiretroviral therapy-related metabolic alterations collectively contribute to a pro-arrhythmic milieu that is not yet fully accounted for in routine clinical care. Key biomarkers such as D-dimer, IL-6, and CRP offer potential not only as indicators of systemic inflammation and coagulopathy but also as tools for cardiovascular risk prediction in this unique population. Despite the insights presented, substantial gaps in evidence remain. Future research must prioritize prospective cohort studies to clarify causal relationships, the integration of inflammatory and coagulative biomarkers into arrhythmia risk algorithms, and ART-specific cardiovascular risk profiling. Personalized strategies for arrhythmia screening and prevention in HIV-positive individuals – guided by both hematologic parameters and guideline-based frameworks – represent a critical next step. Importantly, the current arrhythmia management guidelines from ESC and AHA/ACC/HRS do not specifically account for the HIV-infected population, underscoring the need for more inclusive, multidisciplinary approaches. Bridging this gap will require close collaboration among cardiologists, infectious disease specialists, and hematologists to ensure that the rising burden of non-AIDS-related comorbidities, such as arrhythmias, is addressed with the same rigor as opportunistic infections.

## Data Availability

Not applicable as this a narrative review.

## Research registration unique identifying number (UIN)

Not applicable as this a narrative review.
